# Brief International Cognitive Assessment for MS (BICAMS): international standards for validation

**DOI:** 10.1186/1471-2377-12-55

**Published:** 2012-07-16

**Authors:** Ralph HB Benedict, Maria Pia Amato, Jan Boringa, Bruno Brochet, Fred Foley, Stan Fredrikson, Paivi Hamalainen, Hans Hartung, Lauren Krupp, Iris Penner, Anthony T Reder, Dawn Langdon

**Affiliations:** 1 Masku Neurological Rehabilitation Centre, PO Box 15, Masku, 21251, Finland

## Abstract

An international expert consensus committee recently recommended a brief battery of tests for cognitive evaluation in multiple sclerosis. The Brief International Cognitive Assessment for MS (BICAMS) battery includes tests of mental processing speed and memory. Recognizing that resources for validation will vary internationally, the committee identified validation priorities, to facilitate international acceptance of BICAMS. Practical matters pertaining to implementation across different languages and countries were discussed. Five steps to achieve optimal psychometric validation were proposed. In Step 1, test stimuli should be standardized for the target culture or language under consideration. In Step 2, examiner instructions must be standardized and translated, including all information from manuals necessary for administration and interpretation. In Step 3, samples of at least 65 healthy persons should be studied for normalization, matched to patients on demographics such as age, gender and education. The objective of Step 4 is test-retest reliability, which can be investigated in a small sample of MS and/or healthy volunteers over 1–3 weeks. Finally, in Step 5, criterion validity should be established by comparing MS and healthy controls. At this time, preliminary studies are underway in a number of countries as we move forward with this international assessment tool for cognition in MS.

## Background

Multiple sclerosis (MS) is an inflammatory disease of the central nervous system, causing demyelination and neurodegeneration in most patients [1,2]. As would be expected in such a disease with prominent cerebral pathology, a substantial number [3-5] of MS patients are compromised neuropsychologically. In recently diagnosed or benign course patients, the incidence of cognitive impairment ranges from 20-40% [5,6]. In clinic based samples where secondary progressive course is more common, roughly 50-60% of patients are affected [4].

Neuropsychological (NP) testing provides quantification of cognition, and is used clinically to diagnose impairment and to inform medical and behavioral treatment decisions [7]. Two descriptors, psychometric or neuropsychological tests, are often used inter-changeably to describe the cognitive testing procedures used with MS patients [8]. *Psychometric tests* are standardized, behavioral measures of mental phenomena. They measure many domains of mental function, including psychomotor speed and dexterity, personality or psychopathology [via standardized questionnaires or surveys], intelligence, memory and other aspects of cognitive processing [eg attention, language, executive function].

The term *neuropsychological test* conveys the idea that the psychometric test result is relevant for conclusions pertaining to cerebral function. Neuropsychological tests are used to examine brain-injured patients or to study hypotheses in neuroscience. A deficient neuropsychological test value is often judged to be indicative of cerebral dysfunction. The Wechsler Adult Intelligence Scale (WAIS) [9], is a classic example of a psychometric test of intelligence. It has carefully standardized instructions, scoring criteria, extensive age-based normative data, and information derived from extensive research concerning reliability and validity. It can also be construed as a neuropsychological test [especially nonverbal components] because there are extensive data that show its relationship with cognitive aging, dementia, and other changes in cerebral status.

Psychometric data regarding normal performance, test reliability and the validity of test interpretation are necessary for accurate application of NP testing [10]. Most tests in common use are carefully standardized such that the same instructions, stimuli and marking criteria are used by all examiners. Most often, high test-retest reliability is emphasized in order to avoid error in repeat testing circumstances. Unfortunately, alternate test versions and normative data are not available for some NP tests in all languages and cultures. In addition, many centers lack expertise in psychometrics and NP test interpretation.

The Brief International Cognitive Assessment for MS (BICAMS) initiative was undertaken to recommend a brief, cognitive assessment for MS that is optimized for small centers, with perhaps one or few staff members, who may not have NP training [11]. BICAMS was particularly focused on international use, to facilitate comparison across settings. An expert committee of twelve neurologists and neuropsychologists representing the main cultural groups that have so far contributed extensive data about cognitive dysfunction in MS was convened. The opinions generated from the meeting are published elsewhere [11]. In brief, the panel recommended one particular test with high reliability and good sensitivity, the Rao [12] adaptation of the Symbol Digit Modalities Test (SDMT) [13]. Consensus was also achieved on optimal measures for learning and memory in MS patients, time permitting: the initial learning trials of the second edition of the California Verbal Learning Test (CVLT2) [14] and the revised Brief Visuospatial Memory Test (BVMTR) [15].

In order to facilitate international implementation of the BICAMS assessment, multiple translations are needed, as well as psychometric research to insure the reliability and validity of new test forms. With this in mind, a second conference was held to develop consensus on a BICAMS validation protocol.

## Brief International Cognitive Assessment for MS (BICAMS) description

**Figure 1:**
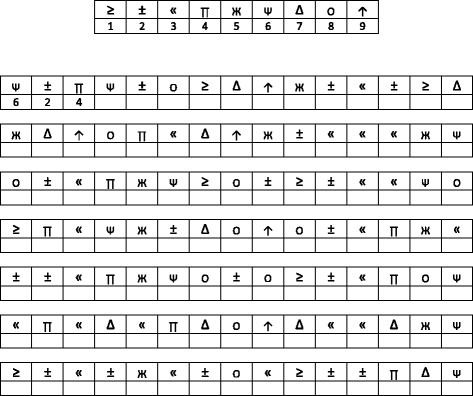
Faux stimuli for Symbol Digit Modalities Test.

The SDMT [13] presents a series of nine symbols, each paired with a single digit in a key at the top of a standard sheet of paper. An adapted version of the test is presented in Figure 1. Patients are asked to voice the digit associated with each symbol as rapidly as possible for 90 sec. There is a single outcome measure – the number correct over the 90 sec time span.

**Figure 2:**
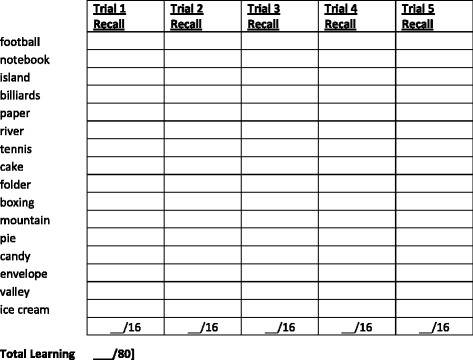
Faux stimuli for California Verbal Learning Test Second Edition.

The auditory/verbal learning test is the CVLT2 [16]. The test begins with the examiner reading a list of 16 words [Figure 2. Patients listen to the list and report as many of the items as possible. There is no instruction as to the order in which items are recalled. After recall is recorded, the entire list is read again followed by a second attempt at recall. Altogether, there are five learning trials. The reader will note that the 16-item list [see faux example in Figure 2 has words that conform to four semantic categories, in this case sports, vegetables, clothes, and tools. Some subjects will recall items in a grouped fashion, and others may recall the list in serial order. There are many variables of recall available in the CVLT2, as a second list is presented, and after 25 min there is a delayed recall trial as well as a yes/no recognition memory task. The BICAMS panel noted that few studies have shown incremental validity with these measures, as the total number of recalled items over the five learning trials is most sensitive [17].

**Figure 3:**
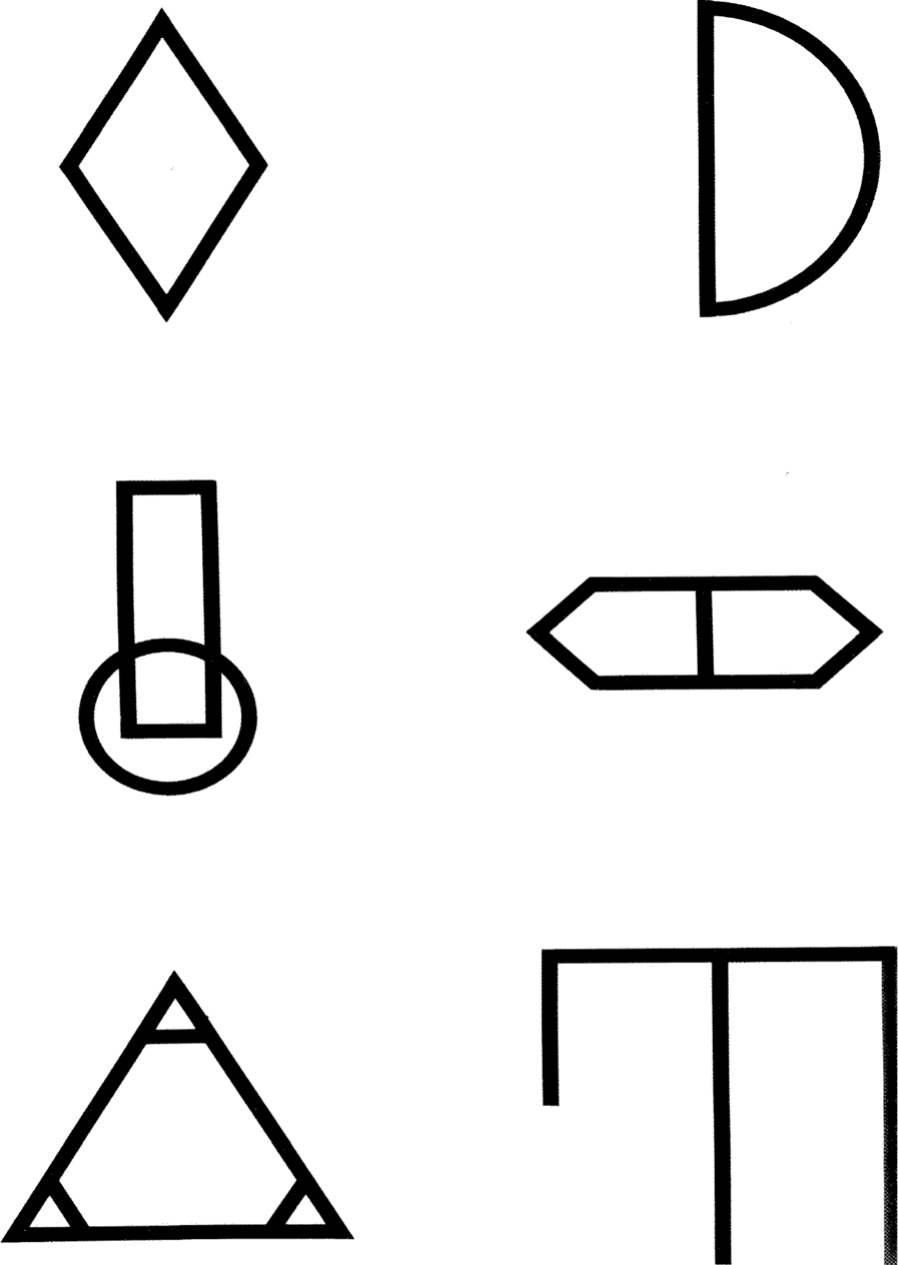
Faux stimuli for Brief Visuospatial Memory Test Revised.

Visual/spatial memory is assessed in BICAMS using the BVMTR [18]. In this test, six abstract designs [Figure 3 are presented for 10 sec. The display is removed from view and patients render the stimuli via pencil on paper manual responses. Each design receives from 0 to 2 points representing accuracy and location. Thus, scores range from 0 to 12. There are three learning trials, and the primary outcome measure is the total number of points earned over the three learning trials. Because there is little evidence that the delayed recall trial adds to discriminant validity in MS [4,19], as in the MATRICS consensus battery [20], only the initial learning trials are recommended for BICAMS.

## Conference process

Having already decided on the core [SDMT] and ancillary memory tests [CVLT2, BVMTR], the focus of the conference was on a BICAMS psychometric validation protocol. The committee reviewed basic psychometric standards from the literature [10,21-26] that are widely accepted for validation of behavioral or psychological outcome measures. Recognizing that economic resources for validation will vary across country and region, the committee discussed priorities for validation, that is, those aspects of research design that would enable empirical appraisal of core psychometrics that may engender confidence and wide application of BICAMS. Following consensus on these central components, it was noted that most of the psychometric evidence relating to the BICAMS tests relies on US samples and discussion shifted to practical matters pertaining to implementation across different languages and countries.

## Psychometric standards

### Standardization

The first step in the development of any test is to ensure that test stimuli and administration procedures have good face validity and consistent stimulus presentation. While this can be a painstaking process during the initial phases of psychometric test development, the work is already done for the tests that were selected for BICAMS. The SDMT, CVLT2 and BVMTR stimuli are well established and are readily mass produced using digital and print technology. Examiners can be easily trained to use standardized instructions, more or less verbatim, to enhance reliability across settings. The BICAMS tests are adequate in this regard.

### Normalization of raw scores

Normative data are of course essential for the clinical application of psychometric, neuropsychological tests. Acquiring normative data can be an expensive endeavor. For example, the recently revised US normative data for BVMTR has a sample size of 588 [27]. These data were fairly recently acquired and are judged by the BICAMS committee to be current.

Normalization data for SDMT are more complicated. The manual based norms date to 1982, raising the spectre of cohort effects [ie gradual shift upward over time] or poor generalizablity to patients and controls in the present day. Benedict et al published normative data on the MACFIMS battery, which includes the SDMT, in 2006 [4] and 2010 [28], using US healthy samples numbering 56 and 120 respectively. These are controlled research studies with applicability largely restricted to clinicians treating MS patients. A potential problem is that normative data from one region [North Eastern USA in this case] may result in interpretive error when applied to raw test scores derived from a different culture, language, region or country.

### Reliability

By reliability we mean the degree to which there is error when using the same instrument across settings, examiners, etc. It is perhaps the most critical psychometric criterion - if the test is unreliable, there is little confidence in the validity of the outcome. Of the various forms of reliability, the panel decided that test-retest reliability has the highest priority and is most relevant for future BICAMS validation. The coefficient of variation can be used in very small samples to determine the extent to which changes in mean values outweigh the variance in test scores [29]. A more valid measure of test-retest reliability is the Pearson correlation coefficient [30]. Most commonly, a brief test-retest interval ranging from 1–3 weeks is employed. For most purposes, r values for test-retest correlation are considered adequate if >0.70 and good if >0.80 [23].

The SDMT has particularly high test-retest reliability. In one US study of MS patients the test-retest r value was 0.97. In a US study repeating the test over six monthly sessions, r values approximated 0.80 for healthy controls and 0.90 for MS patients. Acceptable test-retest reliability [CVLT2 = 0.78; BVMTR = 0.91] was found in a well controlled investigation with US MS patients [31].

### Validity

Rather than accuracy, validity refers to the meaning of a test score. There are multiple aspects of validity in psychometric science. Does a low score, for example, represent the presence of neuropsychological dysfunction, a particular disease state such as MS, a high likelihood of brain atrophy or some other marker of cerebral involvement, or perhaps an increased risk of failing at work? Correlations between test scores and other measures [ie validity coefficient r] and comparing differences in the performance from specified samples [eg, MS vs controls; employed vs disabled MS patients] are common methods of investigation.

The BICAMS committee decided that the most important aspect of validity for clinical purposes is criterion-related validity, most notably differentiating MS patients from healthy controls. All of the BICAMS tests discriminate well with SDMT most often the most sensitive measure in NP batteries [4,19].

A more difficult endeavor is to establish the ecological or predictive validity of a psychometric test [32]. Neuropsychological testing is correlated with a wide range of activities of daily living in MS [33-40], as well as work disability [41-44]. The BICAMS tests are correlated with vocational outcomes [4,45-47] and recently job loss was associated with specific decline of 4–5 points on SDMT [48]. More such research is needed in order to clearly establish thresholds for clinically meaningful changes for the BICAMS measures.

### Alternate test forms

When NP tests are repeated in healthy volunteers or stable MS patients, performance often improves for two reasons: item-specific or task-specific learning [49]. The former refers to the learning of, or memory for, specific test stimuli. For example, on the CVLT2, one might remember specific words from one testing session to the next. Indeed, on a similar test, the 1^st^ trial recall was similar to the delayed recall trial of the same test administered two weeks earlier, but only when the same list was repeated [49]. Test- or task-specific learning refers to the benefit of performing the same behavioral procedure successively, even if the to-be-remembered stimuli are altered. For example, one could possibly learn to label BVMTR figures verbally, a strategy that may carry over to the next session, despite presenting different figures. While both item- and task-specific practice likely play a role in retest effects, in MS, we [31] have found that changing stimuli reduces practice effects on memory tests such as the CVLT2 and BVMTR.

The SDMT was originally published with one test form. Alternate forms were generated by Rao and colleagues [3,12], but in the only work examining inter-form equivalence [50], there was little support for the forms being equivalent. Recently, two new forms were created and found to be equivalent to the standard form [51]. There are two forms for the CVLT2. In the CVLT2 test manual, the normative data are very similar for each form, and the forms yielded similar data in a test-retest within-subjects design [31]. There is strong support for inter-form reliability for the BVMTR [20,31,49,52-54].

## Consensus opinion: the BICAMS validation protocol

The above list of psychometric criteria is not intended to be comprehensive, but the discussed items are essential in the test development process. As we move forward with implementing BICAMS internationally, each of these criteria may be difficult to achieve in other languages and cultures. In this section, we describe the suggested, core validation process, highlighting special considerations for each of the BICAMS measures.

The BICAMS tests were selected, in part, due to extant validation findings and thus it is not surprising that they hold up well to psychometric scrutiny. Table 1 summarizes what we know about the current English versions of BICAMS. Note that the tests are good on the most primary criteria, involving standardization, normalization, test-retest reliability and criterion-related validity. More variable data are available pertaining to alternate forms and predicting clinically meaning changes over time. 

Looking forward, as summarized in **Appendix 1** below, we envisage five steps in future validation protocols in populations for whom English is not the first language.

**Table 1:** Manner in which BICAMS measures meet psychometric criteria in samples with English as a first language

	**SDMT**	**CVLT2**	**BVMTR**
Standardization	Smith 1982 Teat Manual	Delis 2000 Test Manual	Benedict 1997 Test Manual
Normalization	Parmenter 2010. Peer review journal article	Delis 2000 Test Manual	Benedict 2005 Test Manual
Reliability I: Test-Retest	Benedict 2005 r = 0.91	Benedict 2005 r = 0.80	Benedict 2005 r = 0.91
Reliability II: Alternate Form	Rao 1991. Benedict 2012. Good	Delis 2000. Fair	Benedict 1996. Good
Validity I: Criterion Related	Many Studies. Good	Many Studies. Good	Many Studies. Good
Validity II: Clinically Meaningful Change	Morrow 2010. Fair	No or Little Data	No or Little Data

Preliminary work in Step 1 will be needed to maximize standardization while remaining true to the meaning of the original version, where possible. The extant SDMT stimuli are deemed adequate for international use, at least for cultures where Arabic numerals are in common use. One consideration is the pronounciation of numbers which may vary from monosyllabic to polysyllabic utterances [eg one in English and nueve in Spanish], or be simply longer in others [eg üheksa in Estonian]. Rarely, the meaning of the SDMT and BVMTR symbols could become important. These stimuli have little semantic meaning in English but could conceivably have meaning in some cultures. Like the SDMT stimuli, the BVMTR test stimuli are adequate for international application.

The CVLT2 is of course entirely another matter. Here, precise translation necessitates as close approximation of the English words as possible, while maintaining word frequency in the target language, semantic relationships among the target words, orthography, and alike. As noted above, in some languages this could mean a very arduous process. The BICAMS committee agreed that in some countries, another, simple, auditory word-list learning test could replace the CVLT2, provided that the procedure is in the common format – that is reading the list on each learning trial and including at least three learning trials. Some English language examples are the Rey Auditory Verbal Learning Test [55] and the revised Hopkins Verbal Learning Test [56,57].

All BICAMS tests must re-standardize the administration and scoring instructions in the new language. The time required for Step 2 will depend on the specific test and technical support available. The patient instructions for SDMT are brief, and there is minimal instruction necessary for scoring the test in the standardized manner. In contrast, the scoring aspects of the BVMTR manual are quite detailed. Patient responses could be delivered to another party and scored blindly in lieu of translating the entire scoring sections of the manual.

In Step 3, a sample of at least 65 healthy volunteers must be studied with the new BICAMS to develop normative data in the native language. This minimum sample size should provide enough power to detect a medium effect size in a two-group [eg MS vs controls] comparison. Unless a larger sample is available, the normalization sample should be group matched to population studies of MS patients in terms of demographic characteristics. Linear regression approaches can be employed to extend the applicability of the data to demographics that are not fully represented in the database.

Test-retest reliability can be assessed in both patients and controls by a repeat testing session 1–3 weeks after baseline [Step 4]. While both samples are of interest, the panel believes that reliability in MS is more important than in healthy volunteers. In order to assess criterion-related validity [Step 5], the controls must be compared to MS patients, with control for demographics. If Steps 1 and 2 have been completed effectively, all BICAMS variables should discriminate the groups significantly, with d values >0.5. Studies to determine the validity of BICAMS in distinguishing MS populations from healthy controls will need to be carefully constructed, because the criteria of diagnosis of MS adopted may vary among countries [58]. It may be necessary for published data to be segmented to allow comparison with MS samples from other language groups and the BICAMS committee will facilitate this wherever possible.

Finally, other psychometric considerations include inter-rater reliability, alternate forms, and various forms of convergent and discriminant validity. These are not deemed essential, but potentially valuable. For example, does BICAMS predict vocational outcomes? Are low BICAMS scores associated with brain atrophy? Alternate form reliability work has already been commenced by some members of the BICAMS committee.

## Conclusion

The Brief International Cognitive Assessment for MS (BICAMS) initiative was undertaken to recommend a brief, cognitive assessment for MS that can be utilized internationally, in small centers, with perhaps one or few staff members, who may or may not have formal neuropsychological training. Consensus was earlier achieved regarding the BICAMS tests, with special consideration for SDMT, and supplementation by CVLT2 and BVMTR, time permitting. Research is needed to validate BICAMS where English is not the first language. In this article, we have summarized a second consensus opinion which offers a process by which BICAMS can be validated in other languages. Research projects pursuing some of the aims described herein are underway.

## Appendix 1 Recommended Step-by-Step Protocol for BICAMS Validation

▪Step 1, Standardization and Translation of Test Stimuli. For visual stimuli, determine ifthere are any semantic associations to stimuli in the culture or language under consideration. For CVLT2 must match new words on word frequency and appropriate similarity ofmeaning. If these parameters cannot be applied scientifically, then expert review andperformance on test by appropriate participants will be utilized to assess translation.

▪Step 2, Standardization and Translation of Test Instructions. All information from the testmanual necessary for administration and interpretation must be translated, back translated,and checked for errors. Where possible the translated instructions should be validated againstexpected participant performance in terms of accuracy and error profile. Step 4 will alsocontribute to the accuracy of the test instructions.

▪Step 3, Normalization. Large samples of 150 or more healthy persons are needed for dataapplicable to persons of all ages and diverse ethnicity. The minimum sample size is 65healthy volunteers, provided they are group matched on demographics to either a concurrentMS sample, or matched to samples in other published descriptive MS studies. Wherepossible, the distribution of test scores and error profile of the normalization sample shouldbe examined and compared to published distributions from other language groups.

▪Step 4, Test-Retest Reliability. Assessment of this criterion can be achieved by evaluatingan MS and/or healthy volunteer sample on two occasions separated by 1–3 weeks. This is thegold standard separation where the question is only test reliability, c's correlation coefficient >0.70 will usually be required.

▪Step 5, Criterion-Related Validity. This step can be pursued in conjunction with Step 3, inthat an MS sample can be compared to a healthy control group that also serves fornormalization. To determine if a new Italian BVMTR is sensitive to MS disease state, forexample, compare 50 patients to the healthy controls in Step 3. After the study, the investigator adds another 35 healthy volunteers to round out the normalization sample.

## Competing interests

RHBB receives royalties from Psychological Assessment Resources that are in part associated with the Brief Visuospatial Memory Test Revised.

## Authors' contributions

All authors participated in discussion and correspondence to develop this consensus opinion on the topics covered in this article. RHBB is the lead author because he led the development of the manuscript. All authors read and approved the final manuscript.

## Competing financial interests

RHBB has acted as a consultant or scientific advisory board member for Bayer, Biogen Idec, Actelion, and Novartis. He receives royalties from Psychological Assessment Resources, Inc. He has received financial support for research activities from Shire Pharmaceuticals, Accorda and Biogen Idec.

MPA has received research grants and honoraria for serving as speaker at scientific meetings, consultant, and as member of scientific advisory boards from Bayer Pharma AG, Biogen Idec, Merck Serono, Sanofi Aventis, Teva and Novartis.

JB has consulted for Bayer Healthcare and served on speaker bureau for Exencia Pharma Academy.

BB or his institution has received honorarias for speaking at scientific meetings and serving as member of scientific advisory boards for Bayer Pharma, Biogen Idec, Merck Serono, Genzyme, Novartis and Teva and BB's instistution received research grants from Bayer Pharma, Teva, Merck Serono, Novartis, Biogen-Idec, Sanofi-Aventis and ARSEP and Roche.

FF has received honoraria for ad boards and lectures from Biogen, Teva Neuroscience and Novartis. He has received an investigator grant from Bayer Healthcare.

SF has received honoraria for lectures, consultancy and educational activities from Allergan, Bayer, BiogenIdec, MerckSerono, Sanofi, Teva.PH received personal compensation from Bayer Healthcare and Novartis for serving on scientific advisory boards; consulting for Sanofi-Aventis; served on speaker bureau for Bayer Healthcare and Sanofi-Aventis.

PH Dr Hämäläinen received personal compensation from Bayer Healthcare and Novartis for serving on scientific advisory boards; consulting for Sanofi-Aventis; served on speaker bureau for Bayer Healthcare and Sanofi-Aventis.

HPH received honoraria with approval by the Rector of Heinrich-Heine-University from Bayer Healthcare GmbH, Biogen Idec GmbH, Novartis Pharma GmbH, Teva Sanofi Aventis, Hoffman-La Roche and Genzyme Corporation for consulting and speaking at scientific symposia.

LK has served on speaker bureau, scientific advisory boards and/or been a consultant for Teva Neurosciences, BiogenIdec, EMD Serono, Multiple Sclerosis Association of America, Betaseron/Bayer Healthcare Pharmaceuticals, Pfizer, Sanofi-Aventis, Axon Advisors; she has received royalties from Genzyme, ER Squibb & Sons, NMSS, Novartis, MedImmune, Abbott Laboratories, Johnson & Johnson, Roche, Health Professions Conferencing Corp.

IKP has received research grants from Bayer AG Switzerland and the Swiss Multiple Sclerosis Society; has received honoraria forserving as speaker at scientific meetings, consultant, and as member of scientific advisory boards for Actelion, Bayer Pharma AG, Biogen Idec, Merck Serono, Roche, and Teva Aventis.

ATR has no conflicts involving NPsych testing. DWL has received funding for travel to scientific meetings from Bayer Healthcare, Vertex; her institution has received honoraria, consultancy fees, research contracts and sponsorship from Bayer Healthcare, Serono Symposia, Merck-Serono.

DWL has received funding for travel to scientific meetings from Bayer Healthcare, Vertex; her institution has received honoraria, consultancy fees, research contracts and sponsorship from Bayer Healthcare, Serono Symposia, Merck-Serono.

## Pre-publication history

The pre-publication history for this paper can be accessed here:

http://www.biomedcentral.com/1471-2377/12/55/prepub
